# The Treatment of Hypospadias

**Published:** 1876-09

**Authors:** 


The Treatment of Hypospadias.—Prof. Wood, of London, recently showed, at the Medico-Chirurgical Society and King's College Hospital, three cases of hypospadias of a severe character, which had been operated on by his new method of transplantation of the prepuce. In two of the cases the urethra opened at the base of the penis, in front of the scrotum, and in the other about two thirds of the way down.

In all the prepuce was tolerably large, crowning the glans penis like a hood, split below, and without frenum. The method consists of turning up two side flaps, one on each side of the groove which represents the urethra, so as to denude the entire lower surface of the penis, and to remove the contracted cicatricial fibrous tissue which acts so unfavorably in producing a downward curve of the organ during erection. These flaps are turned with the skin surface over the urethral groove, and the edges stitched together by a continuous suture of fine wire, the ends of which are left untwisted and protruding.

A transverse button-hole aperture is then made through the prepuce on the dorsum of the penis, at the attachment to the corona glandis. The glans is then passed through the opening thus made.

The two layers of the prepuce are separated by dissection, so as to permit of the raw surface being laid upon that of the reflected flaps which cover the urethra. The prepuce is finally kept in position by numerous points of thin wire suture.

The patients thus operated on were unable previously to pass the water in a jet, and suffered much inconvenience from the dribbling of the urine over the scrotum, down the legs, and over the clothing. They can now pass the water in a full stream from the end of the newly-formed urethra, the lower part of which is formed by a spout-like projection of the transplanted prepuce.—*Medical and Surgical Reporter.*

